# Inclusion of a multi-ancestry polygenic risk score into a machine learning weight gain prediction model: an observational analysis from the NIH All of Us Research Program

**DOI:** 10.1093/jamiaopen/ooag205

**Published:** 2026-10-23

**Authors:** Kate V Lauer, Lily N Stalter, Bret M Hanlon, Brian W Parks, Tianyuan Lu, Luke M Funk

**Affiliations:** Department of Surgery, University of Wisconsin, Madison, WI 53792-7375, United States; Department of Surgery, University of Wisconsin, Madison, WI 53792-7375, United States; Department of Surgery, University of Wisconsin, Madison, WI 53792-7375, United States; Department of Biostatistics and Medical Informatics, University of Wisconsin, Madison, WI 53792-7375, United States; Department of Nutritional Sciences, University of Wisconsin, Madison, WI 53792-7375, United States; Department of Biostatistics and Medical Informatics, University of Wisconsin, Madison, WI 53792-7375, United States; Department of Population Health Sciences, University of Wisconsin, Madison, WI 53792-7375, United States; Department of Surgery, University of Wisconsin, Madison, WI 53792-7375, United States; Department of Surgery, William S. Middleton Memorial VA, Madison, WI 53705, United States

**Keywords:** obesity, machine learning, All of Us, weight gain prediction, polygenic risk scores

## Abstract

**Objective:**

Obesity is a public health crisis with few effective preventative treatments limited by challenges in identifying who is at high risk of weight gain. To address this gap, our group has developed machine learning weight gain prediction models with modest predictive power. In this study, we aimed to build upon our prior work by incorporating a multi-ancestry polygenic risk score (MAPRS) into a machine learning model and hypothesized that its inclusion would improve weight gain prediction performance.

**Materials and Methods:**

Adults aged 18-70 years with weight measurements 2 years apart were identified within the NIH All of Us Research Program. Participants with a history of cancer, bariatric surgery, or pregnancy were excluded. XGBoost machine learning models were developed using demographics, vitals, laboratory data, obesity-related comorbidities, and MAPRS. The primary outcome was total body weight (TBW) gain of ≥10% over 2 years. Performance was compared using the area under the curves (AUCs).

**Results:**

The cohort consisted of 40 205 individuals (mean [SD] age 51.1[13.5] years; 56.1% female; 48.5% White, non-Hispanic), 3806 (9.5%) of whom gained ≥10% TBW over 2 years. Model performance with and without MAPRS was similar, with AUCs of 0.705 (95% confidence interval [CI] 0.692-0.718) and 0.697 (0.683-0.710), respectively. Baseline age and MAPRS had the highest variable importance. The best net reclassification index was 0.7%, indicating marginal improvements in risk classification with MAPRS.

**Discussion:**

Despite the known hereditary components of obesity and the strong association between the MAPRS and weight gain, incorporating a MAPRS did not substantially enhance model performance.

**Conclusion:**

Further optimization of integrating polygenic risk scores into machine learning weight gain prediction models is needed before they can be applied in the clinical setting.

## Introduction

Obesity, defined as a body mass index (BMI) of ≥30 kg/m^2^, is a public health crisis that affects over 40% of adults in the United States.^1^^,^^2^ While evidence-based treatments such as behavioral management, obesity medications, and bariatric surgery are effective, they have not slowed the rising prevalence of obesity, which is expected to surpass 50% in the United States by 2030.^3^ This has made preventative approaches to obesity essential and has motivated attempts to develop weight gain prediction models. Our group has developed 2 XGBoost machine learning models with similar performance. The first used electronic health record (EHR) and neighborhood data from 24 000 individuals from a single institution,^4^ while the second incorporated EHR and patient survey data from 34 000 individuals in the National Institutes of Health’s All of Us (AoU) dataset.^5^ Both were limited by modest predictive performance (area under the curve [AUC] of 0.68 and 0.71, respectively) and a lack of genomic predictors.

Given that hereditary factors account for as much as 45% of the observed variation in BMI,^6^ it is essential to evaluate the incorporation of genomic predictors into weight gain prediction models. However, BMI is highly polygenic with more than 1100 independent loci^7^^,^^8^ that are individually associated with a change in BMI of 0.01 to 0.09 kg/m^2^.^9^ Polygenic risk scores (PRSs), which quantify an individual’s cumulative genomic risk by combining the effects of single variants, can help in risk stratification for chronic diseases.^10^ Numerous PRS have been developed for BMI, including one containing 2.1 million variants that positively correlated with increased weight, BMI, incidence of severe obesity, and weight gain.^11^ Another found that the risk of increased BMI conferred by a higher PRS could be attenuated with lifestyle modifications.^12^ Our group recently developed a multi-ancestry PRS (MAPRS) using ancestry-specific genome-wide association study (GWAS) meta-analyses that demonstrated improved generalizability across multiple ancestries compared to other scores and captured 7.05% of the variance in BMI.^13^ While PRS show promise as standalone stratification tools for BMI and weight gain, their application as predictors in machine learning models has not been thoroughly studied.

Our objective in this study was to investigate whether incorporating the MAPRS into a machine learning weight gain prediction model would affect model performance. We included the MAPRS as a predictor in an XGBoost machine learning model using the National Institutes of Health AoU dataset, which contains EHR, survey, behavioral, and whole genome sequencing data from over 600 000 racially diverse US adults.^14^ We then compared model performance with and without MAPRS. We hypothesized that the AUC would be higher for the model with MAPRS compared to the same model without.

## Methods

### Machine learning cohort selection

Individuals were selected from the AoU database (version 8), which contained data from 2008 through 2023. Individuals were included if they were aged 18-70 years old and had whole genome sequencing, a baseline BMI classified as overweight or class I obesity (25.0-34.9 kg/m^2^) and weight measurements 2 years apart (±6 months) (Figure 1). This range of baseline BMIs was selected given that individuals considered overweight or class I obesity are at higher risk of developing obesity-related comorbidities with significant weight gain.^15^ For individuals with multiple weight measurements, the 2 most recent weights were used to minimize bias. Individuals were excluded if they had a history of cancer aside from basal or squamous cell skin cancer, bariatric surgery, or pregnancy within 1 year of BMI measurements based on the ICD and SNOMED codes displayed in Table S1, as those diagnoses could confound the observed changes in weight.

**Figure 1.**
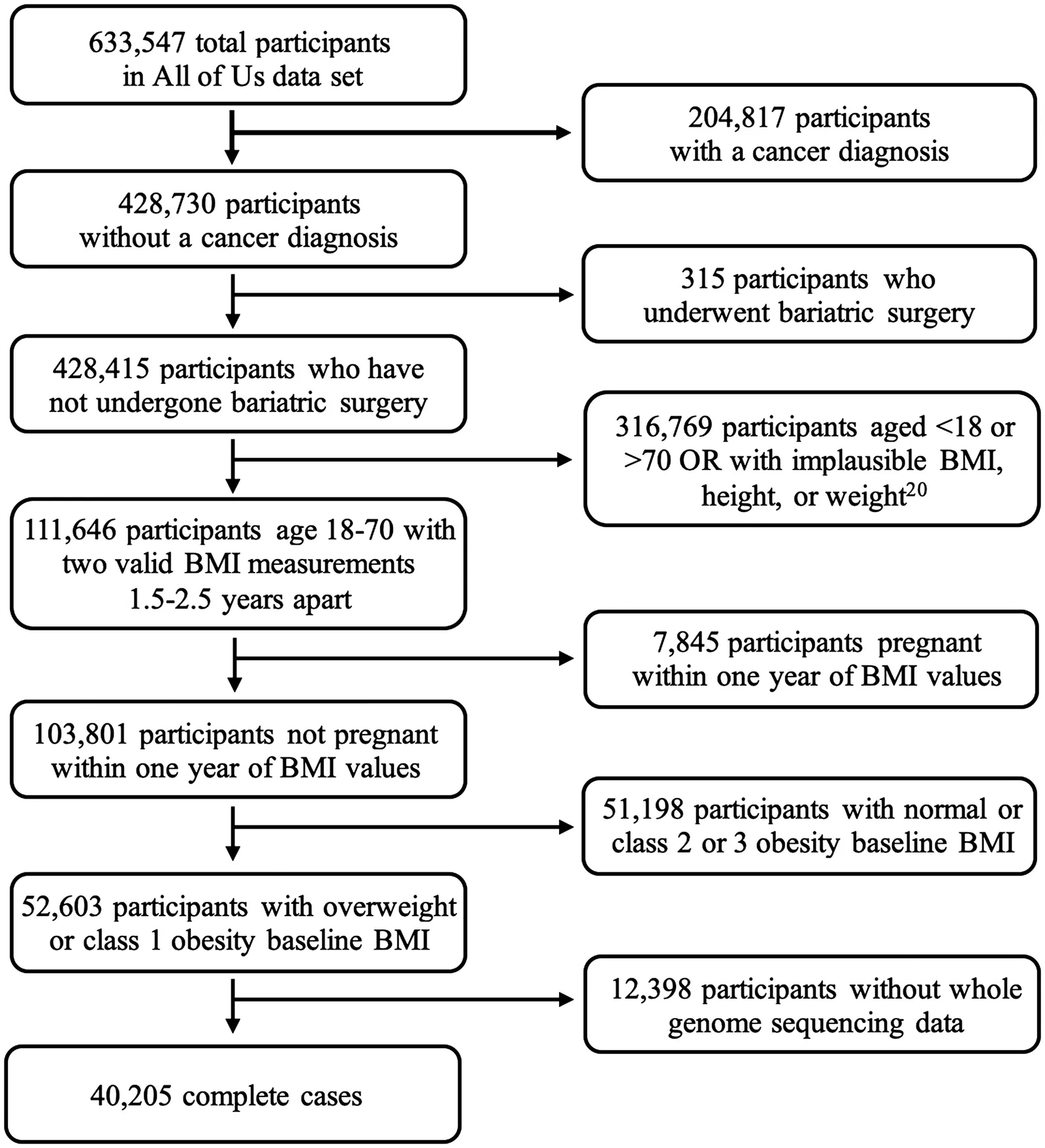
STROBE diagram for All of Us cohort. Abbreviations: BMI, body mass index; OR, odds ratio.

The AoU data were accessed under the Data Use and Registration Agreement with the University of Wisconsin-Madison. No Institutional Review Board was required given that the study did not directly involve human subjects and all data were de-identified. The analytic dataset was created using Python (version 3.10.12), and all machine learning analyses were performed using RStudio (version 4.4.0) and stored in AoU’s cloud-based platform. The Strengthening the Report of Observational Studies in Epidemiology (STROBE) guidelines for case-control studies were reviewed and followed.^16^

### Outcomes

The primary outcome was ≥ 10% total body weight (TBW) gain over 2 years from the baseline weight measurement. This threshold was deemed clinically significant and indicative of high risk based on several prior studies. It is known that both elevated BMI and weight gain are associated with a higher likelihood of developing obesity-related comorbidities.^17–19^ Additionally, 5% weight loss per year has been shown to improve numerous obesity-related comorbidities, including hypertension, type 2 diabetes mellitus (T2DM), and hyperlipidemia,^20–22^ and to increase quality of life.^23^ Based on these studies, ≥ 10% TBW gain over 2 years, which would average to 5% per year, represented both a feasible magnitude of weight gain and one that would increase an individual’s risk of developing obesity-related comorbidities that would negatively impact their long-term health.

### Machine learning predictor variables

The predictors for the machine learning models included demographics, obesity-related comorbidities, vital signs, laboratory data, and PRS. Similar to our prior studies, laboratory and vital sign data were transformed into categorical variables that included a missing category to account for the high frequency of missing values.^4^^,^^5^ Model predictors were compared between individuals who did and did not gain ≥ 10% TBW using T-tests for continuous variables and chi-square tests for categorical factors.

#### Demographics

Demographic predictors included baseline BMI, age at baseline BMI measurement, sex, and insurance type (Medicaid, Medicare, Medicare and Medicaid, none, other, private, and unknown). Race and ethnicity were not included as model predictors to avoid reinforcing social biases and potential confounding with ancestry, which was incorporated into the model with the MAPRS. This approach allowed the model to focus on biological and clinical factors for broader applicability across diverse populations. Implausible height and weight measurements were removed using the algorithm developed by Cheng et al^24^ and applied by our research group in our previous machine learning studies.^4^^,^^5^ If individuals had multiple BMI measurements from a single encounter, the median was used.

#### Obesity-related comorbidities

Obesity-related comorbidities were selected based on well-described associations with obesity and weight gain and included anxiety, cardiovascular disease, chronic pain, depression, gallstones, gastroesophageal reflux disease (GERD), hyperlipidemia, hypertension, nonalcoholic fatty liver disease (NAFLD), obstructive sleep apnea (OSA), osteoarthritis, and T2DM.^15^^,^^25^^,^^26^ All obesity-related comorbidity data were identified by mapping the ICD codes in Table S1 to Concept IDs within AoU.

#### Vital signs and laboratory data

Vital signs included systolic and diastolic blood pressures and heart rate. Laboratory variables included alanine transaminase (ALT), creatinine, glucose, hemoglobin A1C, high-density lipoprotein (HDL) cholesterol, low-density lipoprotein (LDL) cholesterol, and thyroid-stimulating hormone (TSH). Lab values were categorized as normal, abnormal, or missing based on National Health and Nutrition Examination Survey (NHANES) data (Table S2).^27^ For a subset of lab values where additional cutoffs inform disease states, additional descriptive categories were included.

#### Multi-ancestry polygenic risk score

The heritable component of obesity was accounted for within the model using our MAPRS.^13^ This score was developed using ancestry-specific GWAS meta-analyses for individuals of European, African or African American, East Asian, South Asian, and Admixed American ancestry. The MAPRS contained a total of 7913 significant loci (*P* < 5 × 10^−8^) as detailed in Table S3. The MAPRS explained 7.05% of the variance in BMI across all ancestral groups in individuals independent of those in our training and test sets. The variance in BMI explained by the MAPRS was highest for individuals of European ancestry (9.88%) followed by Admixed American (7.79%), South Asian (7.23%), East Asian (6.46%), and African or African American (2.6%). Estimated single nucleotide polymorphism (SNP) heritability, calculated using linkage disequilibrium score regression^28^ with ancestry-matched scores obtained from the Pan-UK Biobank Study,^29^ was highest for individuals of African/African American ancestry (0.2942, SE 0.0207) and lowest for individuals of East Asian ancestry (0.1858, SE 0.0075) (Table S4). Notably, differences in GWAS sample size, linkage disequilibrium structure, allele-frequency distributions, and the correlation of SNP effect estimates across populations can affect polygenic prediction independently of underlying heritability. Thus, higher estimated SNP heritability does not necessarily translate into a higher proportion of phenotypic variance explained by the MAPRS. The MAPRS was calculated for all individuals in our training and test sets and standardized to a mean score of 0 and with a standard deviation of 1.

### Model development and evaluation

An extreme gradient boosting (XGBoost) machine learning method was used to predict ≥ 10% TBW gain over 2 years based on its superior performance in our prior studies.^4^^,^^5^ XGBoost is a gradient-boosted machine well-suited for imbalanced data that utilizes an ensemble of multiple decision trees, with trees added sequentially to the model attempt to correct the classification error of previous trees in the ensemble.^30^ Hyperparameters, including the number of trees, depth of trees, and learning rate, were tuned to maximize the AUC using 10-fold cross-validation on the training set (Table S5). The models were trained using 60% of the data, and predictive performance was validated using the other 40% of the data in the testing set. Stratified sampling was used to ensure that individuals with ≥ 10% TBW gain over 2 years were equally split given the low incidence of the primary outcome.

Model performance for prediction of ≥ 10% TBW gain over 2 years was assessed using AUCs, which were compared using DeLong’s 95% confidence intervals (CIs).^31^ In order to classify the same proportion of the population as high risk between the models with and without MAPRS, predicted values were transformed into percentiles. For example, a cutoff of 0.85 defined the top 15% of individuals in each model as the at-risk group. Accuracy, sensitivity, specificity, positive predictive value (PPV), and negative predictive value (NPV) were calculated for cutoffs of 0.8, 0.85, 0.90, 0.95, and 0.99. F1 scores were calculated using the equation 2(Precision × Recall)/(Precision + Recall), with higher scores indicating higher performance. Net reclassification index (NRI) was calculated to determine the percentage of individuals whose predicted risk category changed after adding MAPRS to the model. A positive NRI would indicate that the model with MAPRS performed better at sorting individuals into appropriate risk groups than the model without MAPRS.

Similar to our previous machine learning studies,^4^^,^^5^ variable importance for outcome prediction was assessed with a model-agnostic approach developed by Williamson et al^32^ that quantifies the difference in predictive performance between the full set of covariates and all covariates except the variable of interest. SHapley Additive exPlanations (SHAP) scores were also calculated and denote the contribution of each variable to outcome predictions with a higher value indicating higher importance.^33^

## Results

### Cohort characteristics

The machine learning cohort consisted of 40 205 participants, as shown in Figure 1 and described in Table 1. The mean baseline age was 51.1 years (standard deviation [SD] 13.5 years). Of the participants, 56.1% were female. Most individuals self-identified as White, non-Hispanic (48.5%) followed by Hispanic (21.7%), Black or African American, non-Hispanic (19.2%), other (5.5%), and Asian, non-Hispanic (2.5%). Of the individuals, 52.5% had European ancestry based on previous genetic clustering analyses,^34^ with African/African American (22.7%), Admixed American (21.1%), East Asian (1.8%), South Asian (1.4%), and Middle Eastern (0.4%) ancestries also represented. The mean baseline BMI was 29.5 kg/m^2^ (SD 2.8) with all individuals classified as either overweight (58%) or having class I obesity (42%). The most common type of insurance was private (40.8%), followed by Medicaid (20.9%) and Medicare (18.0%). The majority of individuals who had vital sign and laboratory data had normal values (Table S6).

**Table 1.** Participant demographics and comorbidity data for entire cohort and by weight gain.

Variable, n (%)	Complete cohort n = 40 205	Gained ≥10% TBW?		
		No n = 36 399 (90.5%)	Yes n = 3806 (9.5%)	*P*-value
**Baseline age (years), mean (SD)**	51.1 (13.5)	51.8 (13.3)	45.2 (13.8)	<.001
**Sex at birth**				<.001
**Female**	22 569 (56.1)	20 024 (55.0)	2545 (66.9)	
**Male**	17 636 (43.9)	16 375 (45.0)	1261 (33.1)	
**Race/ethnicity**				<.001
**White, non-Hispanic**	19 484 (48.5)	17 753 (48.8)	1731 (45.5)	
**Black or African American, non-Hispanic**	7735 (19.2)	6925 (19.0)	810 (21.3)	
**Hispanic**	8741 (21.7)	7875 (21.6)	866 (22.8)	
**Asian, non-Hispanic**	1007 (2.5)	955 (2.6)	52 (1.4)	
**Other**	2211 (5.5)	1956 (5.4)	255 (6.7)	
**Unknown**	1027 (2.6)	935 (2.6)	92 (2.4)	
**Ancestry**				<.001
**European**	21 118 (52.5)	19 216 (52.8)	1902 (50.0)	
**African/African American**	9134 (22.7)	8185 (22.5)	949 (24.9)	
**East Asian**	740 (1.8)	697 (1.9)	43 (1.1)	
**South Asian**	549 (1.4)	508 (1.4)	41 (1.1)	
**Middle Eastern**	173 (0.4)	158 (0.4)	15 (0.4)	
**Admixed American**	8491 (21.1)	7635 (21.0)	856 (22.5)	
**Baseline BMI (kg/m^2^)**				<.001
**Mean (SD)**	29.47 (2.8)	29.49 (2.8)	29.24 (2.8)	
**Overweight**	23 337 (58.0)	20 965 (57.6)	2372 (62.3)	<.001
**Class 1 obesity**	16 868 (42.0)	15 434 (42.4)	1434 (37.7)	
**Insurance type**				<.001
** Private**	16 407 (40.8)	14 922 (41.0)	1485 (39.0)	
** Medicaid**	8406 (20.9)	7333 (20.1)	1073 (28.2)	
** Medicare**	7240 (18.0)	6845 (18.8)	395 (10.4)	
** Medicare and Medicaid**	1675 (4.2)	1480 (4.1)	195 (5.1)	
** Other**	3295 (8.2)	2979 (8.2)	316 (8.3)	
** Missing**	3182 (7.9)	2840 (7.8)	342 (9.0)	
**Obesity-related comorbidities**				
** Anxiety**	6936 (17.3)	6233 (17.1)	703 (18.5)	.038
** Cardiovascular disease**	4740 (11.8)	4361 (12.0)	379 (10.0)	<.001
** Chronic pain**	6847 (17.0)	6206 (17.0)	641 (16.8)	.762
** Depression**	7321 (18.2)	6596 (18.1)	725 (19.0)	.165
** Gallstones**	1334 (3.3)	1200 (3.3)	134 (3.5)	.492
** Gastroesophageal reflux disease**	7943 (19.8)	7276 (20.0)	667 (17.5)	<.001
** Hyperlipidemia**	12 937 (32.2)	12 047 (33.1)	890 (23.4)	<.001
** Hypertension**	18 333 (45.6)	16 776 (46.1)	1557 (40.9)	<.001
** Non-alcoholic fatty liver disease**	2137 (5.3)	1948 (5.4)	189 (5.0)	.331
** Obstructive sleep apnea**	4449 (11.1)	4061 (11.2)	388 (10.2)	.076
** Osteoarthritis**	7094 (17.6)	6533 (17.9)	561 (14.7)	<.001
** Type 2 diabetes mellitus**	7053 (17.5)	6523 (17.9)	530 (13.9)	<.001
**PRS**				<.001
** Lowest decile**	4021 (10.0)	3761 (10.3)	260 (6.8)	
** Middle second-ninth deciles**	32 164 (80.0)	29 130 (80.0)	3034 (79.7)	
** Highest decile**	4020 (10.0)	3508 (9.6)	512 (13.5)	

Abbreviations: BMI, body mass index; PRS, polygenic risk score; SD, standard deviation; TBW, total body weight.

Several differences were observed between those who did and did not gain ≥10% TBW (Table 1). Those who gained ≥10% TBW were younger (45.2 years, SD 13.8 vs 51.8 years, SD 13.3; *P* < .001), and more likely to be female (66.9% vs 55.0%; *P* < .001), overweight (62.3% vs 57.6%; *P* < .001), and insured by Medicaid (28.2% vs 20.1%; *P* < .001). Rates of obesity-related comorbidities were lower in the group that gained ≥10% TBW for all but anxiety, depression, and gallstones. The 2 most common comorbidities for both groups were hypertension and hyperlipidemia. The group that gained ≥10% TBW had a higher proportion of individuals with a MAPRS in the highest decile (13.5% vs 9.6%; *P* < .001).

### Comparison of validated machine learning model performance

Performance of the XGBoost machine learning models with and without MAPRS was compared using AUCs (Figure 2). The models had similar predictive performance. XGBoost with MAPRS had an AUC of 0.705 (95% CI, 0.692-0.718) compared to an AUC of 0.697 (0.683-0.710) for XGBoost without MAPRS. Model accuracy, sensitivity, specificity, PPV, and NPV were similar across all percentile cutoffs with and without MAPRS (Table 2). As expected, accuracy (0.902 for both with and without MAPRS), specificity (0.993), and PPV (0.348) were all highest for the cutoff of 0.99, while sensitivity (0.419 with MAPRS vs 0.416 without) and NPV (0.931 with and without) were highest using 0.8 as the percentile cutoff. NRI was modest across all cutoffs, with the highest value of 0.7% observed at the 0.85 and 0.9 cutoffs, indicating that the addition of MAPRS to the model resulted in, at most, marginal improvements in risk classification (Table 2).

**Figure 2.**
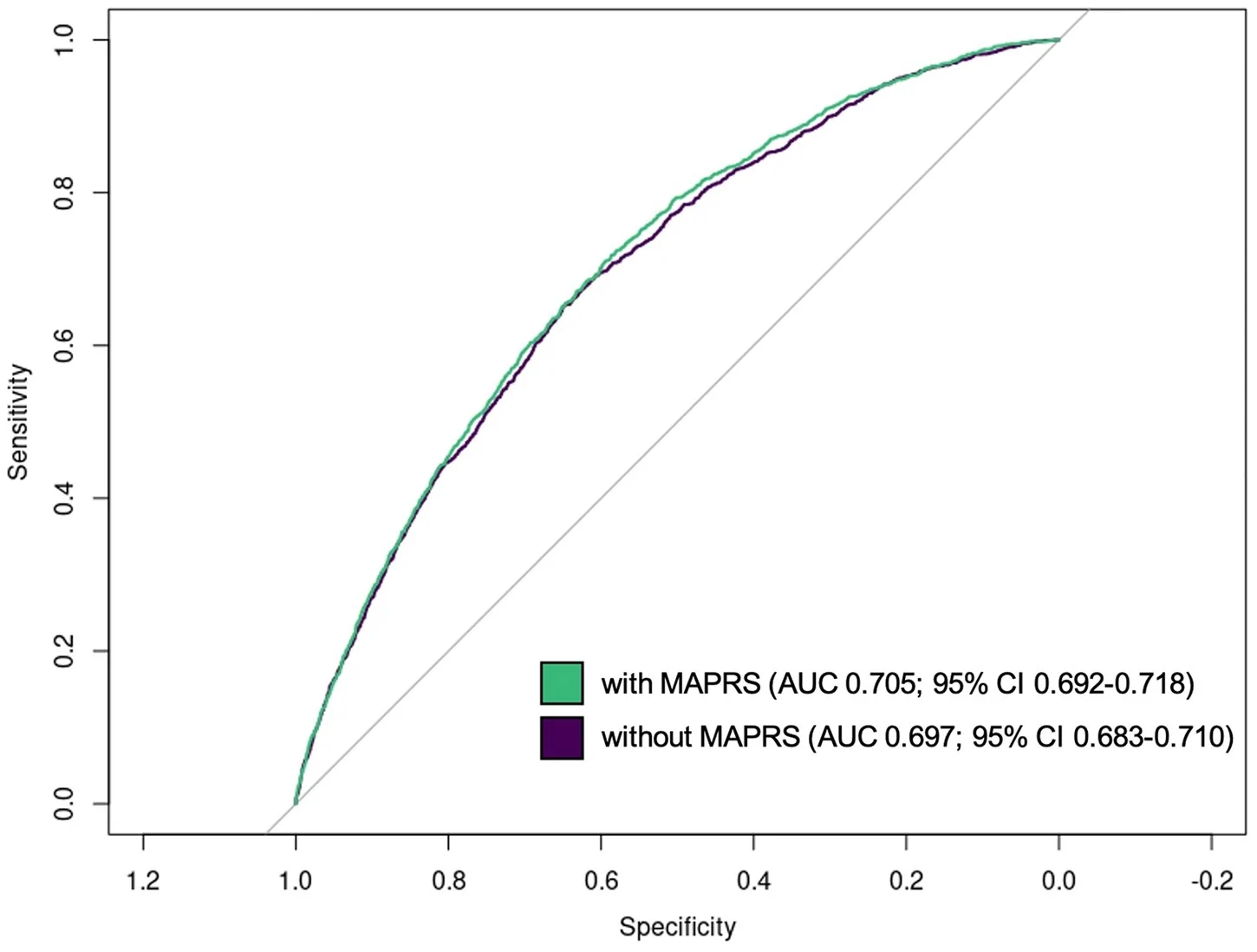
Area under the receiver operating characteristic curves for weight gain prediction models with and without MAPRS. Abbreviations: CI, confidence interval; MAPRS, multi-ancestry polygenic risk score.

**Table 2.** Validated model performance with and without MAPRS.

Model	Percentile cutoff	Accuracy	Sensitivity	Specificity	PPV	NPV	F1 score	NRI
**XGBoost with MAPRS**	0.8	0.785	0.419	0.823	0.198	0.931	0.269	0.003
	0.85	0.820	0.340	0.870	0.214	0.926	0.263	0.007
	0.9	0.851	0.244	0.915	0.231	0.920	0.237	0.007
	0.95	0.881	0.136	0.959	0.257	0.914	0.178	−0.002
	0.99	0.902	0.037	0.993	0.348	0.908	0.067	0.000
**XGBoost without MAPRS**	0.8	0.784	0.416	0.823	0.197	0.931	0.267	
	0.85	0.818	0.333	0.869	0.210	0.926	0.258	
	0.9	0.850	0.237	0.914	0.225	0.920	0.231	
	0.95	0.881	0.138	0.959	0.261	0.914	0.181	
	0.99	0.902	0.037	0.993	0.348	0.908	0.067	

Abbreviations: AUC, area under the receiver operating curve; CI, confidence interval; MAPRS, multi-ancestry polygenic risk score; NPV, negative predictive value; NRI, net reclassification index; PPV, positive predictive value.

### Machine learning model variable importance

Variable importance was analyzed for models with and without MAPRS. Based on Williamson variable importance (WVIM), the most important variable for predicting ≥ 10% TBW gain for both models was baseline age, which increased AUC by approximately 0.06 for both models (Figure 3, Table S7). The model with MAPRS had fewer variables with high importance, but MAPRS was the second most important variable and resulted in a 0.02 increase in AUC. Baseline BMI, sex, heart rate, chronic pain, hypertension, glucose, and depression had higher importance in the model without MAPRS and increased the AUC by 0.01-0.02. Insurance type, most obesity-related comorbidities, and lab values had low variable importance for both models.

**Figure 3.**
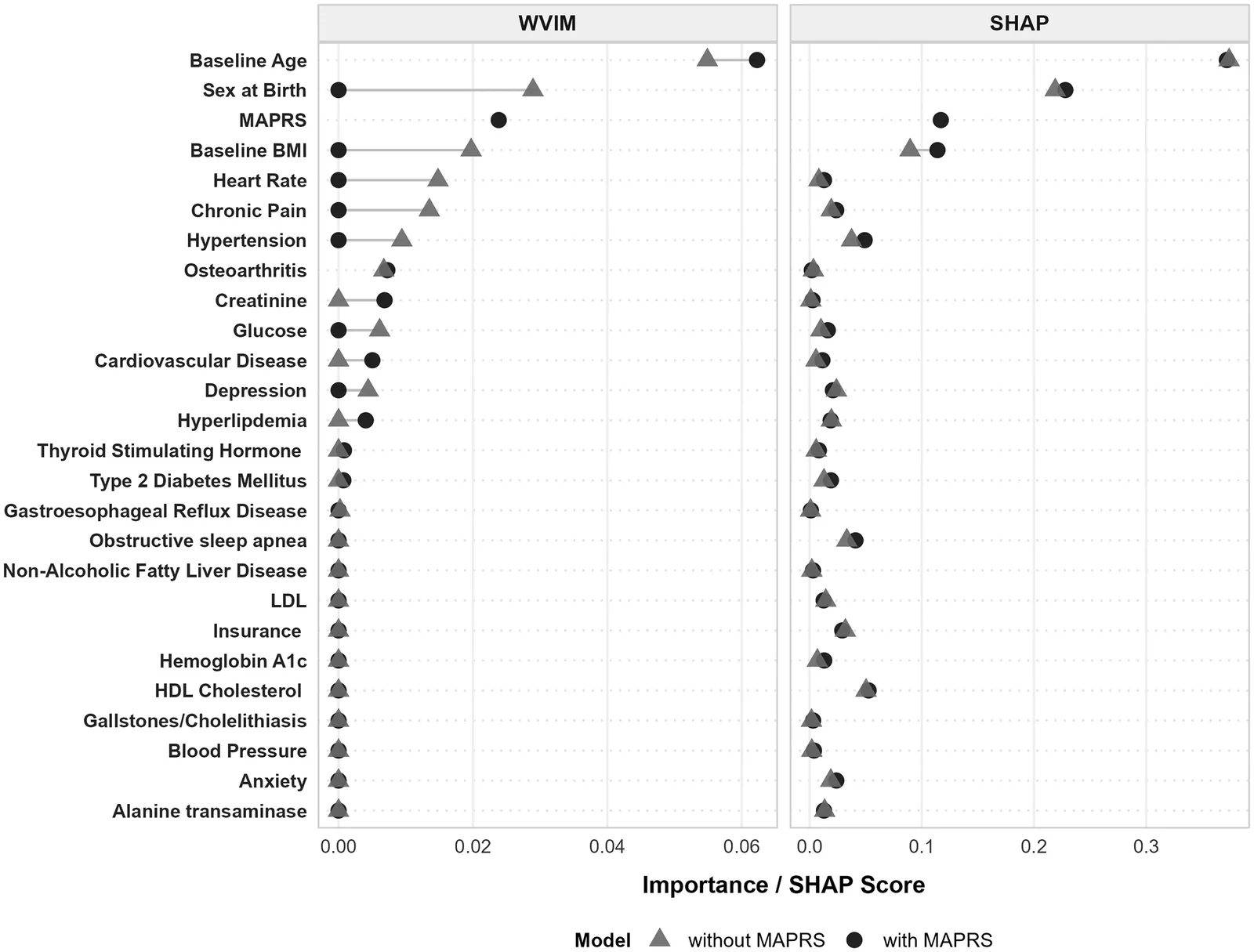
Variable importance for validated weight gain prediction models. Variable importance for each model predictor is shown, with WVIM findings on the left and SHAP scores on the right. Triangles represent the model without MAPRS, while circles represent the model with MAPRS. The WVIM represents the change in AUC when the model was run without the variable of interest. SHapley Additive exPlanations scores denote the contribution of each variable to outcome predictions, with a higher value indicating higher importance. Abbreviations: HDL, high-density lipoprotein; LDL, low-density lipoprotein; MAPRS, multi-ancestry polygenic risk score.

SHapley Additive exPlanations scores demonstrated similar patterns in variable importance. Age and sex at birth were the 2 most important variables for the model with and without MAPRS with scores around 0.4 and 0.2, respectively. In the model with MAPRS, MAPRS and baseline BMI were third and fourth most important scores of 0.1. The rest of the variables had similar scores between models. Overall, variable importance was consistent between the Williamson and SHAP methods.

### Age-restricted sensitivity analysis

A sensitivity analysis was performed including only individuals aged 18-39 (n = 8790) to better assess the influence of participant age on model performance with and without MAPRS. Of the individuals aged 18-39, 1378 (15.7%) gained ≥10% TBW. Area under the curves for the model with and without MAPRS were 0.613 (95% CI, 0.589-0.638) and 0.626 (0.601-0.650), respectively. Performance metrics for the age-restricted models are reported in Table S8. Metrics were similar between the models with and without MAPRS but were lower across all cutoffs compared to the models that were not age restricted. A cutoff of 0.99 again had the highest accuracy (0.842 with MAPRS vs 0.839 without), specificity (0.993 with vs 0.992 without), and PPV (0.417 with vs 0.306 without), while a cutoff of 0.8 had the highest sensitivity (0.307 with vs 0.294 without) and NPV (0.864 with vs 0.862 without). Net reclassification index remained modest across all cutoffs but was higher compared to the model that was not age-restricted, suggesting that the MAPRS had a larger impact on appropriately classifying younger individuals. The highest NRI was 4.3% at the 0.9 cutoff.

## Discussion

Our study aimed to improve the predictive performance of our machine learning weight gain prediction model through a novel approach that integrated genomic data in the form of a MAPRS. Although more individuals who gained ≥10% TBW had a MAPRS in the highest decile and MAPRS had high variable importance within the model, its addition did not improve predictive performance. Model performance remained modest with an AUC of 0.705, and the highest NRI of 0.7% indicated that incorporating the MAPRS resulted in marginal improvements in risk classification.

Despite the known hereditary components of obesity and the strong association between the MAPRS and ≥10% TBW gain across all ancestries, incorporating a MAPRS did not substantially enhance model performance. Integrating PRS into machine learning models that contain other clinical risk factors has not been widely studied, with results indicating that the impact of PRS on model performance varies. One random forest machine learning model for T2DM in individuals of East Asian ancestry generated an increase in AUC of 0.03 from 0.844 to 0.876 after adding an ancestry-specific PRS.^35^ In a second study that used XGBoost to predict Alzheimer’s disease, the impact of PRS on AUC was dependent on the age of individuals included in the models with an increase of 0.04 (0.81 to 0.85) for individuals over 40 years old compared to 0.16 (0.56 to 0.72) for those over 65.^36^ Polygenic risk scores have more frequently been incorporated into risk models based on regression, with observed increases in AUC of 0.01 for major osteopathic fractures (from 0.733 to 0.741),^37^ 0.08 for adult short stature (from 0.812 to 0.896),^38^ and between 0.005 and 0.138 for various types of cancer with subtypes known to have substantial environmental risk factors demonstrating the smallest improvements.^39^ Taken together, the impact of PRS on clinical risk model performance varies significantly and is largest for traits and diseases with high heritability. The lack of improvement in model performance for weight gain prediction seen in this study may be expected based on the heterogeneity in genomic and lifestyle influences that contribute to obesity.

The MAPRS demonstrated high variable importance behind just age and sex at birth yet did not impact model performance. Younger age has consistently been the most important predictor for individuals more likely to gain weight, including in random forest and logistic regression models that predicted obesity in Finnish adults^40^ and our 2 previous machine learning models.^4^^,^^5^ One explanation for the lack of improvement in model performance despite the high variable importance of MAPRS is that shared predictive information between MAPRS and other variables attenuated the net gain in model performance. For example, baseline BMI was highly correlated with MAPRS and had high variable importance in the model without MAPRS. Its variable importance decreased when MAPRS was added, suggesting overlapping predictive information. Of note, associations between the remaining variables and TBW gain may represent confounding or reverse causation rather than causal relationships. In addition, the increase in predictive power may also be limited by the varied heritability of BMI across the lifespan that peaks during puberty and decreases with age.^6^ This is supported by the higher NPI values for the age-restricted model, highlighting how MAPRS has a larger impact on predicting weight gain in younger individuals more susceptible to hereditary drivers. Additionally, the lower AUCs in the age-restricted models suggest that limiting the age range of individuals included in the model reduces cohort heterogeneity in a way that hinders model performance.

Though there was no cumulative effect observed by combining the MAPRS and machine learning model, individuals who gained ≥10% TBW had a higher proportion of MAPRS in the highest decile, reinforcing the ability of the MAPRS to stratify the risk of weight gain over time. One strategy that has been implemented to address this challenge in other chronic diseases is using a stepwise approach that first identifies individuals with high hereditary risk based on PRS followed by utilizing clinical risk models to inform potential interventions. For example, the variance in lithium response prediction for the treatment of bipolar disorder explained by a random forest model increased from 8.1% to 13.7% when individuals were first stratified by PRS.^41^ Similarly, a high PRS for coronary artery disease (CAD) was found to be strongly associated with increased risk of future myocardial infarction. When combined with the Atherosclerotic Cardiovascular Disease Risk Estimator, 20% of individuals were reclassified from borderline to intermediate risk and met criteria for initiating statin therapy.^42^ A recent study also showed that individuals classified as having very high genetic risk (PRS odds ratio >3) for a wide range of cancers and cardiometabolic diseases may reach standard screening thresholds approximately 10 years earlier than average-risk individuals, with projections suggesting that PRS-guided early screening could reduce premature deaths by up to 19%.^34^ Weight gain prediction could benefit from using this type of stepwise screening approach that may reduce the heterogeneity within a population while still effectively incorporating genomic, clinical, and lifestyle risk factors.

Our findings suggest that machine learning weight gain prediction models will require ongoing optimization prior to being integrated into the clinic or community. Further efforts are needed to optimize how and when machine learning and PRSs can be applied to effectively identify those at high risk of weight gain. Future directions to consider include increasing cohort size, developing larger, more robust GWAS for BMI especially for individuals who are not of European ancestry, adopting more sophisticated machine learning methods, testing additional weight gain cutoffs and time periods, and creating ancestry-specific and age-restricted models.

This study has several limitations. First, there may have been inaccuracies in the EHR BMI data in the AoU dataset. This was addressed by excluding implausible values using an algorithm developed by Cheng et al^24^ and used in our previous machine learning studies.^4^^,^^5^ Second, comorbidities were identified using AoU-specific diagnosis codes derived from a list of ICD codes (Table S1). During the data cleaning process, AoU reassigns and combines numerous EHR diagnosis codes into standardized AoU-specific codes that allow researchers to search for diagnoses across diverse datasets. As a result, some comorbidities may not have been accurately captured. Third, the MAPRS was developed using existing GWAS data, which are still disproportionately derived from individuals of European ancestry. Although data from all major ancestry groups were included, the sample sizes for non-European ancestry populations remain much smaller than those for individuals of European ancestry. Future improvements in MAPRS performance will likely depend on continuous efforts to increase the diversity and representativeness of large-scale GWAS. Fourth, given the high variable importance of age and the reduced performance of the age-restricted models, it is possible that the model captures age-related trends rather than heritable factors. Knowing that the heritability of weight gain varies across the lifespan, future directions could include developing independent age-specific models to better account for this confounding. Fifth, our model investigated a single weight gain cutoff of 10% TBW over a 2-year period. It is possible that weight gain prediction may be strengthened by using different cutoffs, which will become easier to study as the AoU database enrolls more patients with more extensive longitudinal data. Lastly, given the observational nature of the study, our models may be susceptible to additional unmeasured confounding.

## Conclusion

Incorporation of a MAPRS into our AoU machine learning weight gain prediction model did not substantially improve predictive performance despite having high variable importance. Model performance remains modest, and further work is needed before these models can be used as a clinical tool for identifying individuals at high risk of clinically significant weight gain.

## Author contributions

Kate V. Lauer (Conceptualization, Formal analysis, Visualization, Writing—original draft, Writing—review & editing), Lily N. Stalter (Conceptualization, Data curation, Formal analysis, Investigation, Methodology, Validation, Visualization, Writing—review & editing), Bret M. Hanlon (Conceptualization, Investigation, Methodology, Writing—review & editing), Brian W. Parks (Conceptualization, Methodology, Writing—review & editing), Tianyuan Lu (Conceptualization, Data curation, Formal analysis, Investigation, Methodology, Software, Validation, Writing—review & editing), and Luke M. Funk (Conceptualization, Formal analysis, Investigation, Resources, Supervision, Writing—original draft, Writing—review & editing)

## Supplementary Material

ooag205_Supplementary_Data

## Data Availability

The data underlying this article will be shared on reasonable request to the corresponding author.
