# Supplementary material for: Inclusion of a multi-ancestry polygenic risk score into a machine learning weight gain prediction model: an observational analysis from the NIH All of Us Research Program
Source: JAMIA Open. 2026 Oct 23;9(5):ooag205. doi: 10.1093/jamiaopen/ooag205 (PMC13690485; doi:10.1093/jamiaopen/ooag205)
Supplement: Supplementary file 1 [file ooag205_supplementary_data.docx]

**SUPPLEMENTARY MATERIALS**

**Table S1: ICD and SNOMED codes used for obesity-related comorbidities and exclusion criteria**

| **Obesity-Related Comorbidity** | **ICD9 Codes** | **ICD10 Codes** |
| --- | --- | --- |
| Anxiety | 300.X | F41.X, F43.0, F43.22, F43.23 |
| Cardiovascular disease | 410.X, 411.0, 411.1, 411.8, 411.81, 411.89, 413.0, 413.1, 413.9, 414.0, 414.00, 414.01, 414.02, 414.03, 414.04, 414.05, 414.06, 414.07, 414.2, 414.3, 414.4, 414.8, 414.9, 429.2, 429.7, 429.71, 429.79, V45.81 | I20.0, I20.1, I20.8, I20.9, I21.X, I22.0, I22.1, I22.2, I22.8, I22.9, I23.0, I23.1, I23.2, I23.3, I23.4, I23.5, I23.6, I23.7, I23.8, I24.0, I24.1, I24.8, I24.9, I25.X, T82.2, T82.21 |
| Cholelithiasis/gallstones | 574.00, 574.01, 574.10, 574.11, 574.20, 574.21, 574.30, 574.31, 574.40, 574.41, 574.50, 574.51, 574.60, 574.61, 575.70, 574.71, 574.80, 574.81, 574.90, 574.91, 575.2, 576.2 | K80.00, K80.01, K80.10, K80.11, K80.12, K80.13, K80.18, K80.19, K80.20, K80.21, K80.30, K80.31, K80.32, K80.33, K80.34, K80.35, K80.36, K80.37, K80.40, K80.41, K80.42, K80.43, K80.44, K80.45, K80.46, K80.47, K80.50, K80.51, K80.60, K80.61, K80.62, K80.63, K80.64, K80.65, K80.66, K80.67, K80.70, K80.71, K80.80, K80.81, K82.0, K83.1 |
| Chronic pain | 338.2, 338.29, 338.4 | F45.4, F45.41, F45.42, G89.2, G89.29, G89.4 |
| Depression | 300.4, 309.0, 309.1, 311 | F32.X, F33.X, F34.X, F39.X, F43.21, F43.23 |
| Gastroesophageal reflux disease | 530.1, 530.10, 530.11, 530.12, 530.13, 530.19, 530.8, 530.81, 530.85, 787.1 | K20.0, K20.8, K20.9, K21.0, K21.9, K22.7, K22.70, K22.71, K22.710, K22.711, K22.719, R12 |
| Hyperlipidemia | 272.0, 272.1, 272.2, 272.3, 272.4 | E78.0, E78.00, E78.01, E78.1, E78.2, E78.3, E78.4, E78.5 |
| Hypertension | 401.X, 402.X, 403.X, 404.X, 405.X | I10, I11.X, I12.X, I13.X, I16.X |
| Nonalcoholic fatty liver disease (NAFLD) | 571.8, 571.9 | I10, I11.X, I12.X, I13.X, I16.X, K75.81, K76.0 |
| Obstructive sleep apnea | 327.2X, 780.51, 780.53, 780.57, 786.03 | G47.3X, R06.81 |
| Osteoarthritis | 715.X | M15.X, M16.X, M17.X, M18.X, M19.X |
| Pre-diabetes | 790.21, 790.22, 790.29 | R73.01, R73.02, R73.09, R73.9 |
| Type 2 diabetes | 250.X | E11.X |
| **Exclusion Criteria** | **ICD9 Codes** | **ICD10 Codes** |
| Cancer | 140.0-165.X, 170.0-172.9, 174.0-176.9, 179.0-209.X, 230.0-231.9, 233.0-239.X | C0.0-C43.9, C4A.X, C45.0-C96.9, D00-D03.9, D05.00-D09.X, D32.X, D37-D49.X |
| Pregnancy | 630-679.14 | O0.0-O9A53, Z33.X, Z34.X, Z3A.X, Z37.X, Z38.X, Z39.0, A34.X |
| Bariatric surgery | **SNOMED Codes** 430715008, 427074001, 173747005, 708983005, 414574004, 426738005 | |

“X” represents any possible number. All ICD and SNOMED codes were mapped to All of Us concept IDs using the Dataset Builder.

**Table S2: Cut-offs for laboratory and vital sign data**

| **Lab Value** | **Definition** | **Cohort Range** | **Implausibility Cut-off** | **Implausibility Cut-off Justification** |
| --- | --- | --- | --- | --- |
| Alanine transaminase (U/L) | Normal: < 55  Abnormal: ≥ 55 | 3-1311 | N/A | None needed given the upper value is clinically plausible |
| Creatinine (mg/dL) | Normal: < 1.2  Abnormal: ≥ 1.2 | 0.17-29.79 | >73 | Yang X, Gao M, Miao M, Jiang C, Zhang D, Yin Z, Ni Y, Chen J, Zhang J. Combining combretastatin A4 phosphate with ginsenoside Rd synergistically inhibited hepatocellular carcinoma by reducing HIF-1α via PI3K/AKT/mTOR signalling pathway. J Pharm Pharmacol. 2021 Mar 4;73(2):263-271. doi: 10.1093/jpp/rgaa006. |
| Glucose (mg/dL) | Normal: < 100  Pre-diabetes: 100-125  Diabetes: ≥ 126 | 50-1071 | <50  >2,656 mg/dl | Upper limit implausibility cut-off: Guinness World Record 2008 |
| HDL (mg/dL) | Normal: ≥ 60  Abnormal: < 60 | 5.7-161 | <5  >189 | [NHANES laboratory data (August 2021 data release)](https://wwwn.cdc.gov/nchs/nhanes/search/DataPage.aspx?Component=Laboratory) |
| A1C (%) | Normal: ≤ 5.6  Pre-pre-diabetes: 5.7-6.4  Diabetes: ≥ 6.5 | 3.2-16.1 | <2.8  >16.2 | [NHANES laboratory data (May 2021 data release)](https://wwwn.cdc.gov/nchs/nhanes/search/DataPage.aspx?Component=Laboratory) |
| LDL (mg/dL) | Normal: ≤ 129  Borderline high: 130-159  Abnormal ≥ 160 | 14-332 | <14  >359 | [NHANES laboratory data (October 2021 data release)](https://wwwn.cdc.gov/nchs/nhanes/search/DataPage.aspx?Component=Laboratory) |
| TSH (uIU/mL) | Normal: 0.32 - 4  Hypothyroid: > 4  Hyperthyroid: < 0.32 | 0.035-92.018 | <0.035  >99.564 | [NHANES laboratory data (October 2015 data release)](https://wwwn.cdc.gov/nchs/nhanes/search/DataPage.aspx?Component=Laboratory) |
| **Vitals** | **Definition** | **Cohort Range** | **Implausibility Cut-off** | **Implausibility Cut-off Justification** |
| Systolic blood pressure (mmHg) | Pre-hypertension: either SBP ≥ 130 but < 140 OR DBP ≥ 80 but < 90 | 45-241 | Exclude if no systolic BP  Exclude >300 mmHg | [NHANES 2015-2016 Data Documentation, Codebook, and Frequencies](https://wwwn.cdc.gov/Nchs/Nhanes/2015-2016/BPX_I.htm) |
| Diastolic blood pressure (mmHg) | Pre-hypertension: either SBP ≥ 130 but < 140 OR DBP ≥ 80 but < 90 | 2-149 | Exclude if no diastolic BP  Exclude if >systolic | [NHANES 2015-2016 Data Documentation, Codebook, and Frequencies](https://wwwn.cdc.gov/Nchs/Nhanes/2015-2016/BPX_I.htm) |
| Heart rate (BPM) | Normal: 60-99  Bradycardia: < 60 Tachycardia: ≥ 100 | 40-165 | Exclude <40  Exclude if >predicted maximum heart rate (220 – age) | <https://www.cdc.gov/physical-activity-basics/about/index.html?CDC_AAref_Val=https://www.cdc.gov/physicalactivity/basics/measuring/heartrate.htm> |

**Table S3: Ancestry-informed** **MAPRS data sources**

| **Ancestry** | **GWAS Data Sources** | **Number of Participants** | **Number of Independent Variants** |
| --- | --- | --- | --- |
| European | MVP  UK Biobank  GIant Consortium  FinnGen | 424,221  419,163  322,154  500,348 | 5,875 |
| African/African American | MVP  UK Biobank  Uganda Genome Resource  Durban Diabetes Study  Diabetes Case Control Study  Africa American Diabetes Mellitus Study | 118,993  6,545  6,197  1,114  1,478  5,187 | 945 |
| East Asian | MVP  UK Biobank  BioBank Japan  Taiwan Biobank | 6,384  2,693  163,835  92,615 | 702 |
| South Asian | East London Genes and Health  UK Biobank | 34,408  8,646 | 79 |
| Admixed American | MVP | 57,793 | 312 |

*MVP: Million Veterans Program; UK: United Kingdom; Admixed: individuals whose ancestry is derived from two or more of the above groups due to migration within the last several hundred years* Significance for association between individual variants and BMI defined as P <5 x 10^-8^.

**Table S4: Estimated SNP heritability by ancestry**

| **Ancestry** | **SNP Heritability** | **Standard Error** |
| --- | --- | --- |
| African/African American | 0.2942 | 0.0207 |
| Admixed American | 0.2853 | 0.0208 |
| East Asian | 0.1858 | 0.0075 |
| European | 0.1869 | 0.0054 |
| South Asian | 0.2005 | 0.0172 |

SNP heritability was estimated using linkage disequilibrium score regression.^28^ Ancestry-matched linkage disequilibrium scores were obtained from the Pan-UK Biobank study.^29^

**Table S5: Considered and top performing parameters for machine learning models**

| **Model** | **Model R Package** | **Tuning Parameter** | **Considered Values** | **Top Parameter without MAPRS** | **Top Parameter with MAPRS** |
| --- | --- | --- | --- | --- | --- |
| XGBoost | xgboost | nrounds | 50 to 1000 by 50 | 950 | 600 |
|  |  | max_depth | 1, 2, 3, 4, 5, 6 | 2 | 2 |
|  |  | eta range | 0.01, 0.015, 0.025, 0.05, 0.1, 0.3 | 0.01 | 0.025 |
|  |  | gamma | 0, 0.05, 0.1, 0.5, 0.7, 0.9, 1 | 0 | 0.1 |
|  |  | colsample_bytree | 0.2, 0.3, 0.4, 0.6, 0.8, 1 | 0.4 | 0.3 |
|  |  | min_child_weight | 1, 2, 3,4 | 3 | 2 |
|  |  | subsample | 0.5, 0.75, 1 | 0.75 | 0.75 |

Considered and top performing parameters for XGBoost machine learning model. A tuning grid was not used for alpha or lambda. Instead, tunelength=20 was used, allowing the algorithm to select different default values. All models were tuned in caret package.

**Table S6: Participant vital sign and laboratory data**

| **Variable, n (%)** | **Machine Learning Cohort (n=40,205)** |
| --- | --- |
| **Heart Rate (bpm)** |  |
| Normal | 28,099 (69.9) |
| Bradycardia | 2,744 (6.8) |
| Tachycardia | 1,657 (4.1) |
| Missing | 7,705 (19.2) |
| **Blood pressure (mmHg)** |  |
| Normal | 30,141 (75.0) |
| Pre-hypertension | 185 (0.5) |
| Hypertension | 7,048 (17.5) |
| Missing | 2,831 (7.0) |
| **Alanine transaminase (u/L)** |  |
| Normal | 16,241 (40.4) |
| Abnormal | 1,194 (3.0) |
| Missing | 22,770 (56.6) |
| **Creatinine (mg/dL)** |  |
| Normal | 22,868 (56.9) |
| Abnormal | 3,127 (7.8) |
| Missing | 14,210 (35.3) |
| **HDL Cholesterol (mg/dL)** |  |
| Normal | 4,444 (11.1) |
| Low | 14,642 (36.4) |
| Missing | 21,119 (52.5) |
| **LDL Cholesterol (mg/dL)** |  |
| Normal | 11,905 (29.6) |
| Borderline-High | 2,605 (6.5) |
| High | 1,177 (2.9) |
| Missing | 24,518 (61.0) |
| **Glucose (mg/dL)** |  |
| Normal | 14,288 (35.5) |
| Pre-diabetes | 7,307 (18.2) |
| Diabetes | 4,450 (11.1) |
| Missing | 14,160 (35.2) |
| **A1C (%)** |  |
| Normal | 5,467 (13.6) |
| Pre-diabetes | 3,555 (8.8) |
| Diabetes | 2,712 (6.7) |
| Missing | 28,471 (70.8) |
| **Thyroid Stimulating Hormone (TSH) (uiu/mL)** |  |
| Normal | 7,684 (19.1) |
| Hypothyroid | 583 (1.5) |
| Hyperthyroid | 237 (0.6) |
| Missing | 31,701 (78.8) |

**Table S7: Williamson variable importance (WVIM) and SHAP (SHapley Additive exPlanations) values**

| **Feature** | **WVIM** | | **SHAP** | |
| --- | --- | --- | --- | --- |
|  | **With MAPRS** | **Without MAPRS** | **With MAPRS** | **Without MAPRS** |
| Baseline Age | 0.062278492 | 0.054883729 | 0.372 | 0.374 |
| Alanine transaminase | 0 | 0 | 0.0128 | 0.0134 |
| Anxiety | 0 | 0 | 0.0238 | 0.0189 |
| Gallstones/Cholelithiasis | 0 | 0 | 0.00308 | 0.00168 |
| Blood Pressure | 0 | 0 | 0.00387 | 0.00195 |
| Baseline BMI | 0 | 0.019734228 | 0.114 | 0.0896 |
| Cardiovascular Disease | 0.005035322 | 0 | 0.0114 | 0.00566 |
| Creatinine | 0.00684649 | 0 | 0.0026 | 0.00108 |
| Depression | 0 | 0.004407895 | 0.0208 | 0.0239 |
| Type 2 Diabetes Mellitus | 0.000721677 | 0 | 0.019 | 0.0128 |
| Gastroesophageal Reflux Disease | 0 | 0.000216728 | 0.00109 | 0.000876 |
| Glucose | 0 | 0.006119413 | 0.0162 | 0.0101 |
| HDL Cholesterol | 0 | 0 | 0.0526 | 0.0503 |
| Heart Rate | 0 | 0.014802317 | 0.0128 | 0.00823 |
| Hemoglobin A1c | 0 | 0 | 0.013 | 0.00693 |
| Hyperlipdemia | 0.004050589 | 0 | 0.0188 | 0.0194 |
| Insurance | 0 | 0 | 0.0291 | 0.0319 |
| LDL | 0 | 0 | 0.0125 | 0.0144 |
| Hypertension | 0 | 0.009424064 | 0.0491 | 0.0374 |
| Non-Alcoholic Fatty Liver Disease | 0 | 0 | 0.003 | 0.00197 |
| Obstructive sleep apnea | 0 | 0 | 0.0409 | 0.0332 |
| Osteoarthritis | 0.007260324 | 0.006736526 | 0.00195 | 0.00336 |
| Chronic Pain | 0 | 0.013509922 | 0.0237 | 0.0193 |
| MAPRS | 0.023836884 | - | 0.117 | - |
| Sex at Birth | 0 | 0.028936966 | 0.228 | 0.219 |
| Thyroid Stimulating Hormone | 0.000807455 | 0 | 0.00823 | 0.00583 |

**Table S8: Validated model performance for age-restricted sample of individuals aged 18-39 with and without MAPRS**

| **Model** | **Cut point** | **Accuracy** | **Sensitivity** | **Specificity** | **PPV** | **NPV** | **F1** | **NRI** |
| --- | --- | --- | --- | --- | --- | --- | --- | --- |
| XGBoost with MAPRS | 0.80 | 0.739 | 0.307 | 0.820 | 0.240 | 0.864 | 0.269 | 0.015 |
|  | 0.85 | 0.765 | 0.229 | 0.865 | 0.239 | 0.858 | 0.234 | 0.009 |
|  | 0.90 | 0.797 | 0.172 | 0.913 | 0.270 | 0.856 | 0.210 | 0.043 |
|  | 0.95 | 0.820 | 0.085 | 0.956 | 0.267 | 0.849 | 0.129 | 0.017 |
|  | 0.99 | 0.842 | 0.027 | 0.993 | 0.417 | 0.846 | 0.051 | 0.009 |
| XGBoost without MAPRS | 0.80 | 0.735 | 0.294 | 0.817 | 0.230 | 0.862 | 0.258 |  |
|  | 0.85 | 0.763 | 0.221 | 0.863 | 0.231 | 0.856 | 0.226 |  |
|  | 0.90 | 0.786 | 0.136 | 0.907 | 0.213 | 0.850 | 0.166 |  |
|  | 0.95 | 0.815 | 0.071 | 0.954 | 0.222 | 0.847 | 0.107 |  |
|  | 0.99 | 0.839 | 0.020 | 0.992 | 0.306 | 0.845 | 0.037 |  |

*AUC: area under the receiver operating curve; CI: confidence interval; PPV: positive predictive value; NPV: negative predictive value; NRI: net reclassification index*
